# CSNK1A1, KDM2A, and LTB4R2 Are New Druggable Vulnerabilities in Lung Cancer

**DOI:** 10.3390/cancers13143477

**Published:** 2021-07-12

**Authors:** Elisabetta Sauta, Francesca Reggiani, Federica Torricelli, Eleonora Zanetti, Elena Tagliavini, Giacomo Santandrea, Giulia Gobbi, Silvia Strocchi, Massimiliano Paci, Giovanna Damia, Riccardo Bellazzi, Davide Ambrosetti, Alessia Ciarrocchi, Valentina Sancisi

**Affiliations:** 1 Laboratory of Translational Research, Azienda USL-IRCCS di Reggio Emilia, 42122 Reggio Emilia, Italy; elisabetta.sauta01@universitadipavia.it (E.S.); Francesca.Reggiani2@ausl.re.it (F.R.); Federica.Torricelli@ausl.re.it (F.T.); giulia.gobbi@ausl.re.it (G.G.); silvia.strocchi@ausl.re.it (S.S.); alessia.ciarrocchi@ausl.re.it (A.C.); 2 Department of Electrical, Computer and Biomedical Engineering, University of Pavia, 27100 Pavia, Italy; riccardo.bellazzi@unipv.it; 3 Pathology Unit, Azienda USL-IRCCS di Reggio Emilia, 42122 Reggio Emilia, Italy; eleonora.zanetti@ausl.re.it (E.Z.); elena.tagliavini@ausl.re.it (E.T.); Giacomo.Santandrea@ausl.re.it (G.S.); 4 Clinical and Experimental Medicine Ph.D. Program, University of Modena and Reggio Emilia, 41100 Modena, Italy; 5 Thoracic Oncology Unit, Azienda USL-IRCCS di Reggio Emilia, 42122 Reggio Emilia, Italy; massimiliano.paci@ausl.re.it; 6 Laboratory of Molecular Pharmacology, Department of Oncology, Istituto di Ricerche Farmacologiche Mario Negri IRCCS, 20156 Milan, Italy; giovanna.damia@marionegri.it; 7 Department of Pharmacy and Biotechnology (FaBit), University of Bologna, 40126 Bologna, Italy; davide.ambrosetti@unibo.it

**Keywords:** lung cancer, CRISPR/Cas9 screening, CSNK1A1, KDM2A, LTB4R2

## Abstract

**Simple Summary:**

The main histological subtypes of lung cancer are small-cell lung cancer (SCLC) and non-small-cell lung cancer (NSCLC). NSCLC is further subdivided into squamous-cell carcinoma (SCC) and adenocarcinoma (AD). Despite the recent introduction of innovative therapies, lung cancer is still the first cause of cancer-related human death, indicating that the discovery of new therapeutic targets is still a compelling need for this disease. In the present work, we performed a functional genomics analysis on different lung cancer histotypes, combining data derived from different omics resources with in vitro validation. Through this approach, we identified and validated CSNK1A1, KDMA2, and LTB4R2 as new druggable vulnerabilities in lung cancer. These results open new possibilities for the development of innovative therapies for lung cancer patients.

**Abstract:**

Lung cancer is the leading cause of cancer-related human death. It is a heterogeneous disease, classified in two main histotypes, small-cell lung cancer (SCLC) and non-small-cell lung cancer (NSCLC), which is further subdivided into squamous-cell carcinoma (SCC) and adenocarcinoma (AD) subtypes. Despite the introduction of innovative therapeutics, mainly designed to specifically treat AD patients, the prognosis of lung cancer remains poor. In particular, available treatments for SCLC and SCC patients are currently limited to platinum-based chemotherapy and immune checkpoint inhibitors. In this work, we used an integrative approach to identify novel vulnerabilities in lung cancer. First, we compared the data from a CRISPR/Cas9 dependency screening performed in our laboratory with Cancer Dependency Map Project data, essentiality comprising information on 73 lung cancer cell lines. Next, to identify relevant therapeutic targets, we integrated dependency data with pharmacological data and TCGA gene expression information. Through this analysis, we identified CSNK1A1, KDM2A, and LTB4R2 as relevant druggable essentiality genes in lung cancer. We validated the antiproliferative effect of genetic or pharmacological inhibition of these genes in two lung cancer cell lines. Overall, our results identified new vulnerabilities associated with different lung cancer histotypes, laying the basis for the development of new therapeutic strategies.

## 1. Introduction

Despite remarkable research efforts and the groundbreaking introduction of targeted therapy and immune therapy, lung cancer is still the leading cause of cancer-related death. Indeed, the prognosis is still poor, with a five year survival rate of only 17% [1]. Lung cancer is a heterogeneous disease, classified into two main types based on histological patterns: small-cell lung cancer (SCLC) and non-small-cell lung cancer (NSCLC). SCLC represents about 13% of cases, whereas NSCLC is further subdivided in various subtypes, the most prevalent of which are adenocarcinoma (AD) (50%) and squamous-cell carcinoma (SCC) (22%) [2,3]. Lung cancer subtypes differ in genetic alterations, carrying distinct sets of mutations and rearrangements [3]. In AD, several drivers of genetic alterations have been identified and mutations on EGFR gene and gene fusions involving ALK or ROS1 genes have been targeted with specific small-molecule inhibitors [4]. Although driver mutations have also been characterized for SCC and SCLC, the current standard treatment for these patients is platinum-based chemotherapy in combination with immune checkpoint inhibitors [5,6].

A substantial breakthrough in the treatment of lung cancer is represented by the introduction of immune checkpoint inhibitors, which are now used for all three main lung cancer subtypes [4,7]; however, only a fraction of patients show a sustained response to treatment, and reliable biomarkers predicting response to therapy have not been identified yet [7].

For these reasons, a deep molecular characterization of lung cancer is still required to identify new or overlooked targets that will allow the development of novel strategies, including single or combination therapy approaches. In this context, an innovative perspective is moving the focus from mutated oncogenes to the non-mutated gene dependency of cancer cells. This notion, also called “non-oncogene addiction”, is based on the finding that many cancer types rely for their survival—more than normal cells—on genes that are not classical oncogenic drivers. As a consequence, cancer cells are more sensitive than normal cells to the specific inhibition of those non-oncogenes, creating an intriguing therapeutic window [8]. 

The identification of non-oncogene addictions requires genome-wide approaches able to portray pathway interactions and functional consequences of genetic perturbations. On the other hand, the possibility to translate the findings of genomic screenings into clinical practice is often hampered by the lacking targetability of screening hits and poor correspondence between in vitro models and patient characteristics.

In a previous studied, we defined an integrative functional genomics approach, combining in vitro non-oncogene dependency data within a large collection of lung cancer cell lines, gene druggability information, patients’ gene expression data, and in vitro validation. Through this approach, we identified and validated the EGLN1 gene as a new pro-oncogenic factor and druggable dependency specifically associated with the KRAS-mutated lung AD setting [9]. In this follow-up paper, using a similar approach, we extended our analyses to cell lines derived from different lung cancer histotypes, including AD, SCLC, and SCC. We identified and validated a set of novel druggable non-oncogene vulnerabilities preferentially related to different lung cancer histotypes; dependency on KDM2A is associated with AD and SCLC, dependency on CSNK1A1 is associated with AD, and dependency on LTB4R2 is associated with SCC.

## 2. Materials and Methods

### 2.1. CRISPR/Cas9 Genomic Screening

The human GeCKOv2 CRISPR knockout pooled library in lentiGuide-Puro plasmid was a gift from Feng Zhang (Addgene # 1000000049) [10]. The screening was conducted as already described [11]. 

### 2.2. CERES Score Generation

To make our CRISPR/Cas9 screening of the A549 cell line data comparable to the DepMap A549 dependency data (19Q2 release), we normalized raw data from the two semi-libraries GeCKOv2 A and GeCKOv2 B using the CERES computational method, which estimates gene dependency while correcting for the copy number effect [12]. The sgRNA sequences of the semi-libraries were aligned to the hg19 reference genome using bowtie2 (2.3.5.1 version) [13] and samtools [14]; the resulting alignments were then mapped to gene coding sequences obtained from CCDS database (https://www.ncbi.nlm.nih.gov/projects/CCDS/CcdsBrowse.cgi, accessed on 12 December 2019). Together with copy number data downloaded from the CCLE data portal (https://portals.broadinstitute.org/ccle, accessed on 12 November 2019), we fit the CERES algorithm, which was implemented in R language and is available at https://github.com/cancerdatasci/ceres, accessed on 10 September 2019. The hyperparameter λ was set to 0.681 as suggested by the authors [12] for the GeCKO library. The resulting CERES scores for semi-libraries A and B were then compared to A549 DepMap data to evaluate the dependency value similarities of common genes. Then, to proceed for downstream analyses, we integrated RNA sequencing data (19Q2 release) obtained from the DepMap data portal in order to focus only on expressed genes that were present in all three libraries. To highlight dependency genes within this common ground, we defined a dependency threshold (*D*), as shown below in Equation (1):

(1) $$\frac{D=\sum_{i=1}^{n}\left(CERES\right)}{n}+2\sigma$$

where *n* is the number of common essential genes (*i*) defined by the DepMap project, for which the CERES scores were considered as the average plus two standard deviations in order estimate the dependency threshold (*D*).

### 2.3. Histotype-Based Analysis

We downloaded the essentiality data from the DepMap repository for all lung cancer cell lines (n = 73), the information for which was integrated with histotype classification of the considered lines, namely AD, SCLC, and SCC cell lines, respectively. To select dependency genes underlying each histological type, we applied our D cut-off to retain only those genes with a CERES score below this threshold in at least 90% of the AD, SCLC, or SCC cell line. To further evaluate the biological relevance of the resulting gene lists for the examined histotypes, we performed a pathway enrichment analysis through the ClueGO app [15] within the Cytoscape software platform (3.7.1 version) [16], using Reactome and KEGG pathways as references. Only the pathways that were significantly enriched with a Benjamini–Hochberg adjusted *p* value ≤ 0.05 were considered and graphically represented. 

### 2.4. Druggability Analysis

The essentiality gene lists deriving from the histotype-based analysis were submitted to the Drug–Gene Interaction Database (https://www.dgidb.org/, accessed on 15 January 2020) [17]. The resulting drugs were filtered to keep only those reporting an “inhibitor”, “antagonist”, or “modulator” mode of action indication. Each list of gene–drug interactions was manually curated to group the identified drugs into the following categories: “approved for lung cancer”; “approved for other cancer conditions”; “in clinical trials for cancer conditions”; “approved or in clinical trials for non-cancer conditions”, “pre-clinical data on target”. In addition to the literature search, we took advantage of pharmacological data available in DrugBank (https://go.drugbank.com/, accessed on 25 January 2020) [18] and Clinical Trials (https://clinicaltrials.gov, accessed on 25 January 2020) databases.

### 2.5. TCGA Analysis

AD and SCC RNA seq data were downloaded from TCGA database and analyzed using the R TCGA biolinks package (project = “TCGA-LUAD” (AD) and “TCGA-LUSC” (SCC); data.category = “TranscriptomeProfiling”; data.type = “Gene Expression Quantification”; workflow.type = “HTSeq-FPKM”). SCLC RNA seq data extracted from the study “U Cologne, Nature 2015” [19], were downloaded from cBioPortal repository (https://www.cbioportal.org/, accessed on 15 April 2021) and analyzed using R software.

### 2.6. Patient Specimens and Gene Expression Analysis

Fresh-frozen tissue samples from lung cancer patients were retrieved from the Biobank of AUSL-IRCCS di Reggio Emilia. Informed consensus was obtained from all patients. This study was authorized by the local ethical committee (Comitato Etico dell’Area Vasta Emilia Nord) and conducted according to the Helsinki Declaration. Patients’ features are summarized in Supplementary Table S3. Total RNA was extracted from surgical samples conserved in liquid nitrogen. For each patient, RNA was extracted from lung AD and surrounding healthy lung tissue using TRIzol (Thermo Fisher, Waltham, MA, USA) and analyzed as already described [20,21]. GUSB and cyclophilin A were used as reference genes. Primer sequences are shown in Supplementary Table S4.

### 2.7. Cell Cultures and Treatments

A549 and NCI-H23 lung cancer cell lines were obtained from Dr. Massimo Broggini (IRCCS—Istituto di Ricerche Farmacologiche Mario Negri, Milan, Italy). The HEK293T cell line was purchased from ATCC (LGC Standards, Sesto S. Giovanni, Italy). All cell lines were sub-cultured in RPMI-1640 medium (Thermo Fisher) supplemented with 10% fetal bovine serum (FBS, Euroclone, Milan, Italy) and antibiotics at 37 °C and 5% CO_2_. All cell lines are authenticated through SNP profiling by Multiplexion Gmbh (Heidelberg, Germany) and are routinely checked for mycoplasma contamination. Cell lines were treated for 72 h with a range of concentrations of D4476 (MedChem Express, Monmouth Junction, NJ, USA), daminozide (MedChem Express, Monmouth Junction, NJ, USA), or LY255283 (Santa Cruz Biotechnology, Dallas, TX, USA), then dissolved in DMSO and further diluted in complete medium. DMSO alone was used as treatment control. Cell growth was monitored using the Incucyte^®^ S3 live cell imaging system (Essen Biosciences Inc, Ann Arbor, MI, USA) and EC50 curves were generated using Incucyte^®^ software (2020B version, Essen Biosciences Inc, Ann Arbor, MI, USA).

### 2.8. Generation of KO Cell Lines

Sequences of two sgRNAs were selected for each target gene from independent CRISPR KO libraries (GeCKOv2 Library and Brunello Library) [10,22] and cloned into the pLKO5.sgRNA.EFS.GFP plasmid. The pLKO5.sgRNA.EFS.GFP was a gift from Benjamin Ebert (Addgene plasmid # 57822, Addgene, Watertown, MA, USA) [23]. A non-targeting sgRNA was used as the negative control. NSCLC cell lines expressing Cas9 were infected as previously described to obtain the KO cell lines of target genes [11]. An Alt-R^®^ genome editing detection kit (#1075931, IDT, Skokie, IL, USA) was used to confirm on-target CRISPR event efficiency. Sequences of sgRNAs target sequences and of primers used for Alt-R analysis are shown in Supplementary Table S4.

### 2.9. Competition Assay

NSCLC cells with stable Cas9 expression were infected with a reduced multiplicity of infection (MOI) of lentiviral particles to obtain 50% of infected target cells. The pLKO5.sgRNA.EFS.GFP plasmid was used as the lentiviral backbone, carrying a single sgRNA for each target gene. GFP expression was quantified to monitor the proliferation of the infected vs. non-infected cells. Cells were checked through a BD FACSCanto™ II Cell Analyzer (BD, Franklin Lakes, NJ, USA) every 3 days for 21 days. Each experiment was carried out in triplicate with independent infections and with 2 different sgRNAs for each target gene.

### 2.10. Western Blot

Western blot analysis was performed as previously described [24]. Briefly, total cell lysate was obtained with PLB Buffer (Promega, Madison, WI, USA) added with a protease inhibitor cocktail (bimake.com, Munich, Germany). Soluble proteins were separated from debris by 10 min centrifugation at 12,000 rpm and quantified with a Bradford Protein Assay (Bio-Rad Laboratories S.r.l., Milan, Italy). The following primary antibodies were used, following the manufacturer’s instruction: rabbit anti-KDM2A 1:500 (#A301-475A-T, Bethyl Laboratories, Montgomery, TX, USA), rabbit anti-CSNK1A1 1:500 (#orb382647, Biorbyt, Cambridge, UK), rabbit anti-LTB4R2 1:1000 (Biorbyt, Cambridge, UK), mouse anti-beta actin 1:5000 (#A2228, Sigma-Aldrich, St. Louis, MO, USA).

### 2.11. Statistical Analysis

The statistical evaluation of our patient gene expression analysis and of competition assays was performed using GraphPad Prism software (GraphPad, San Diego, CA, USA). Statistical significance was determined using Student’s *t* test. Each experiment was performed in triplicate.

## 3. Results

### 3.1. Identification of Lung Cancer Dependencies Integrating Genome-Wide CRISPR/Cas9 Screening Data

**Figure 1:**
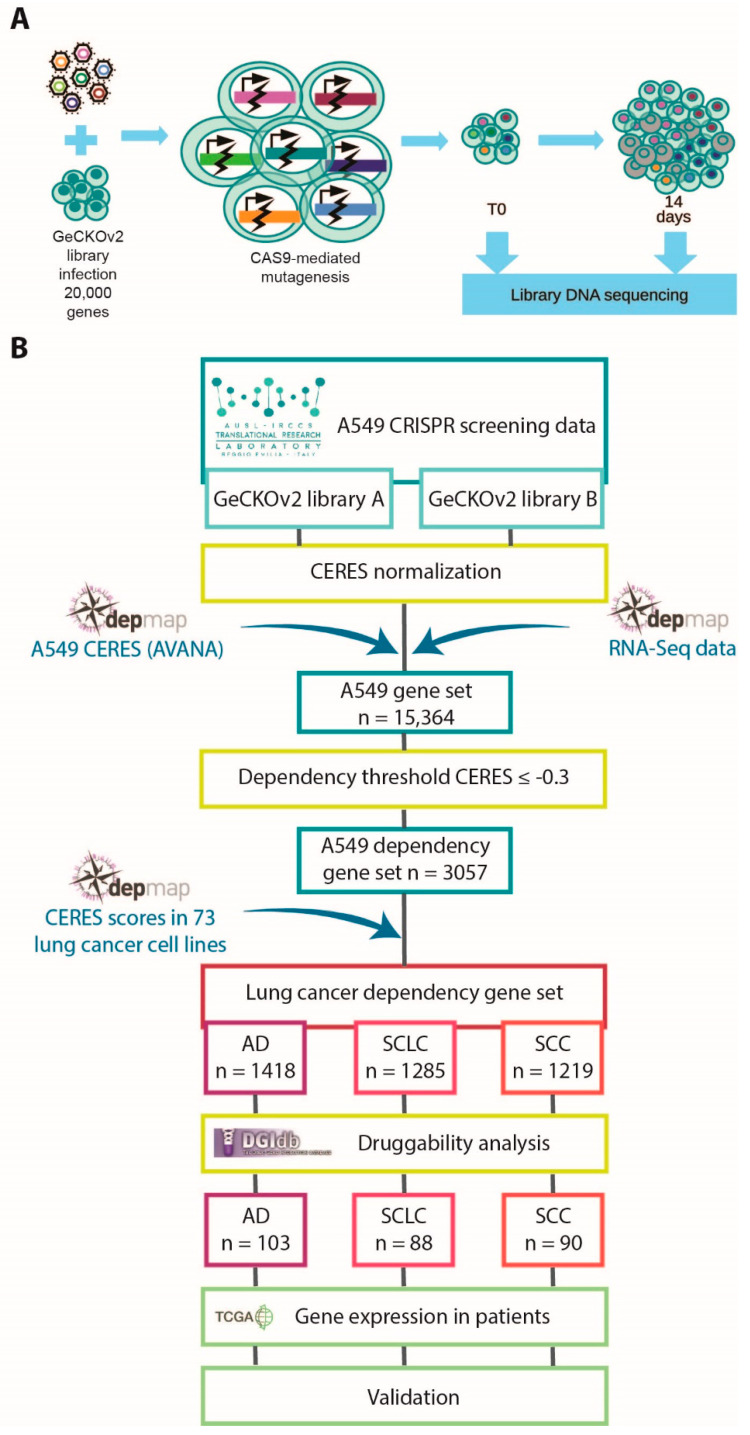
Overview of the performed analyses: (**A**) schematic representation of the CRISPR/Cas9 screening procedure; (**B**) schematic representation of the integrated bioinformatics and functional genomics approach that we applied to identify novel drug target candidates in lung cancer.

We aimed to identify genes that are required for lung cancer cell proliferation and that can be used as new therapeutic targets. We performed CRISPR/Cas9 screening in the A549 cell line, derived from lung adenocarcinoma (Figure 1A and Supplementary Video S1). We used the GeCKOv2 genomic library, targeting nearly 20,000 genes in the human genome and containing 6 sgRNAs for each gene, distributed into two semi-libraries, GeCKOv2 A and GeCKOv2 B [10]. 

**Figure 2:**
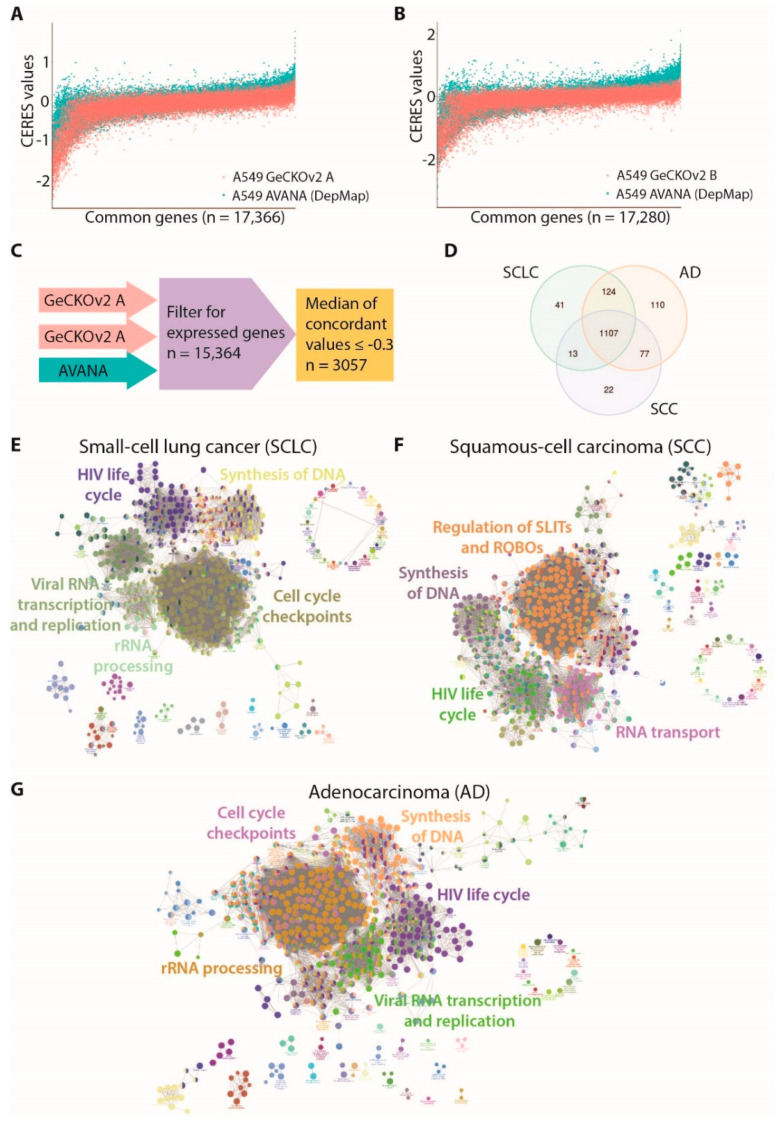
Identification of genes and pathways essential for lung cancer main histotypes: (**A**) comparison of CERES scores along all common genes between AVANA library (DepMap) and GeCKOv2 sub-library A in A549 cells; (**B**) comparison of CERES scores along all common genes between AVANA library (DepMap) and GeCKOv2 sub-library B in A549 cells; (**C**) schematic representation of filtering strategy used to identify the A549 dependency genes; (**D**) Venn diagram summarizing the number of genes whose CERES score resulted ≤−0.3 (D threshold) in at least 90% of cell lines for each histotype (AD: adenocarcinoma; SCLC: small-cell lung cancer; SCC: squamous-cell carcinoma; (**E**) network representation of significantly enriched pathways for the SCLC dependency gene set (n = 1285); (**F**) network representation of significantly enriched pathways for the SCC dependency gene set (n = 1219); (**G**) network representation of significantly enriched pathways for the AD dependency genes set (n = 1418).

To confirm the validity of our screening results, we retrieved different omics resources and exploited a data-integration-based approach, combining dependency data of a large collection of lung cancer cell lines from the Broad Institute’s Achilles project and available on the DepMap portal (https://depmap.org/portal/, accessed on 12 November 2019) [12,25] with information on gene–drug interactions (https://www.dgidb.org/, accessed on 15 January 2020) [17], gene expression data in lung cancer patients available on The Cancer Genome Atlas (TCGA) repository (https://www.cancer.gov/tcga, accessed on 27 November 2019) [26], and in vitro assay validation. A flowchart of the integrative procedure is shown in Figure 1B. First, we took advantage of DepMap dependency data [12], a database including genome-wide mutational, gene expression, and dependency information for 73 lung cancer cell lines, comprising AD, SCC, and SCLC cell lines. The DepMap consortium developed the CERES algorithm to compare essentiality screening data among different cell lines, taking into account copy-number-amplified regions that may lead to false-positive results [12]. We applied the CERES algorithm to our screening data, obtaining a CERES score for each gene in A549 cells. Since we performed two fully independent screenings with the two semi-libraries, we kept the two datasets separated and compared them with the corresponding DepMap data obtained using the AVANA library. Comparisons of CERES scores among the three libraries indicated substantial similarity (Figure 2A,B). To improve the robustness of our analysis, we exploited DepMap RNA sequencing data to exclude non-expressed genes. Next, for each expressed gene presenting sgRNAs in all the three libraries, we calculated the median of the concordant CERES values. To select essential genes, we established a threshold (D) for CERES scores at −0.3, representing the mean value plus two standard deviations of CERES scores for common essential genes in A549 cells (Figure 2C). From this analysis, we ended up with a robust list of 3057 dependency genes in the A549 cell line, identified by taking into account both our data generated with the two GeCKOv2 semi-libraries and DepMap data generated with AVANA library.

### 3.2. Identification of Dependencies Preferentially Associated with Lung Cancer Histotypes

We compared the results obtained in A549 cells with the DepMap collection of 73 lung cancer cell lines, reflecting patients’ histological and mutational complexity. The CERES score datasets for 73 lung cancer cell lines were downloaded from the DepMap portal. We grouped different cell lines according to their histotype and we identified the genes that were dependencies in at least 90% of either AD, SCLC, or SCC cell lines. Out of 3057 A549-specific dependency genes, 1418, 1285, and 1219 were dependencies in AD, SCLC, and SCC cell lines, respectively. This analysis identified three partially overlapping sets of essentiality genes, with specific lists for the three histotypes and a common core of 1107 genes, which is shared by all groups (Figure 2D). To understand the biological processes underlying the different lung cancer histotypes, we performed a pathway enrichment analysis. The synthesis of DNA and RNA metabolic pathways were significantly enriched in essential genes for all three histotypes. Cell cycle checkpoints and cell-cycle-related processes were particularly enriched in AD and SCLC cell lines, while pathways involved in regulation of Roundabout receptors (ROBOs) and SLIT ligands were a specific dependency for the SCC cell line (Figure 2E–G and Supplementary Table S1).

### 3.3. Identification of CSNK1A1, KDM2A, and LTB4R2 as Druggable Dependencies in Lung Cancer

**Figure 3:**
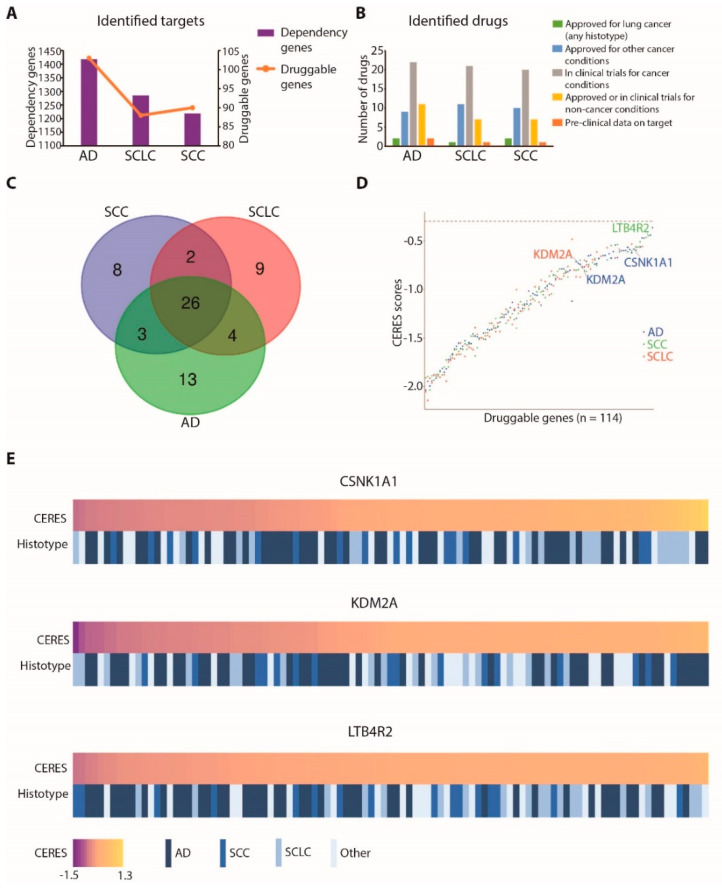
Identification of druggable dependencies in lung cancer: (**A**) bar plot summarizing identified targets and druggable targets in lung cancer cell lines for each considered histotype (bars and left Y axis represent all dependency genes, while the orange line and right Y axis represent the subgroup of druggable genes for each histotype; AD: adenocarcinoma; SCLC: small-cell lung cancer; SCC: squamous-cell carcinoma); (**B**) bar plot representing the number of identified drugs and the status of each drug for each lung cancer histotype; (**C**) Venn diagram summarizing the number of druggable dependency genes for each histotype (AD: adenocarcinoma; SCLC: small-cell lung cancer; SCC: squamous-cell carcinoma); (**D**) median CERES score values in the cell lines of each histotype for the identified druggable genes (values for KDM2A in AD and SCLC cell lines, for CSNK1A1 in AD cell lines, and for LTB4R2 in SCC cell lines are labelled); (**E**) heatmap showing lung cancer cell lines ordered by dependency on CSNK1A1, KDM2A, or LTB4R2 genes, expressed as CERES scores. For each cell line, the histotype is indicated.

To translate our results into possible therapeutic strategies, we focused on “druggable” genes that can be directly targeted with chemical compounds. To this end, we crossed the dependency genes lists with the Gene–Drug Interaction Database (https://www.dgidb.org/, accessed on 15 January 2020) [17]. From this analysis, we identified 103 dependencies and pharmacologically druggable genes in AD, 88 in SCLC, and 90 in SCC. Notably, most of our identified drug–gene interactions have been previously characterized and the matched drugs have been approved or are under evaluation in clinical trials for cancer treatment, strongly supporting the validity of our approach (Figure 3A–C and Supplementary Table S2) [27,28].

Next, we focused our attention on dependency genes that are chemically targetable but that still require biological validation as lung cancer vulnerabilities. In this category, we selected three genes: CSNK1A1 (casein kinase 1 Alpha 1), KDM2A (lysine demethylase 2A), and LTB4R2 (leukotriene B4 receptor 2). CSNK1A1 was a druggable dependency gene specifically associated with AD, KDM2A was associated with both AD and SCLC, whereas LTB4R2 was associated with SCC cell lines (Figure 3D). Across different lung cancer cell lines, these genes showed a variable extent of dependencies, ranging from highly addicted to not-affected cell lines, as depicted in Figure 3E.

**Figure 4:**
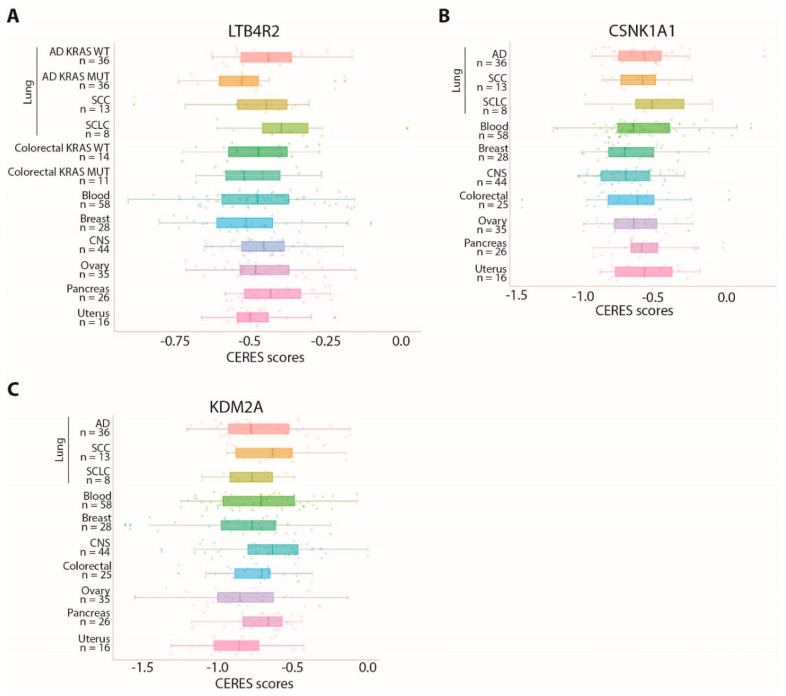
Cell lines derived from different tumor types show variable dependency on CSNK1A1, KDM2A, or LTB4R2: (**A**–**C**) comparison of CERES score distributions for the LTB4R2 (**A**), CNK1A1 (**B**), and KDM2A (**C**) genes in cell lines derived from different tumor tissues. The number of cell lines considered for each cancer type is indicated on the Y axis.

Notably, LTB4R2 was also identified in our previous work as a dependency gene specifically associated with the KRAS-mutated background in lung AD [9]. As shown in Figure 4A, the CERES scores for this gene were comparable in SCC cell lines and KRAS-mutated AD cell lines, with two SCC cell lines showing remarkably low scores.

We verified whether these genes are shared dependencies with other cancer types, comparing CERES scores in cancer cell lines derived from different tissues. All these genes are also dependencies in other cancer types, although with variable scores (Figure 4A–C).

### 3.4. CSNK1A1, KDM2A, and LTB4R2 Are Overexpressed in Lung Cancer Patients

**Figure 5:**
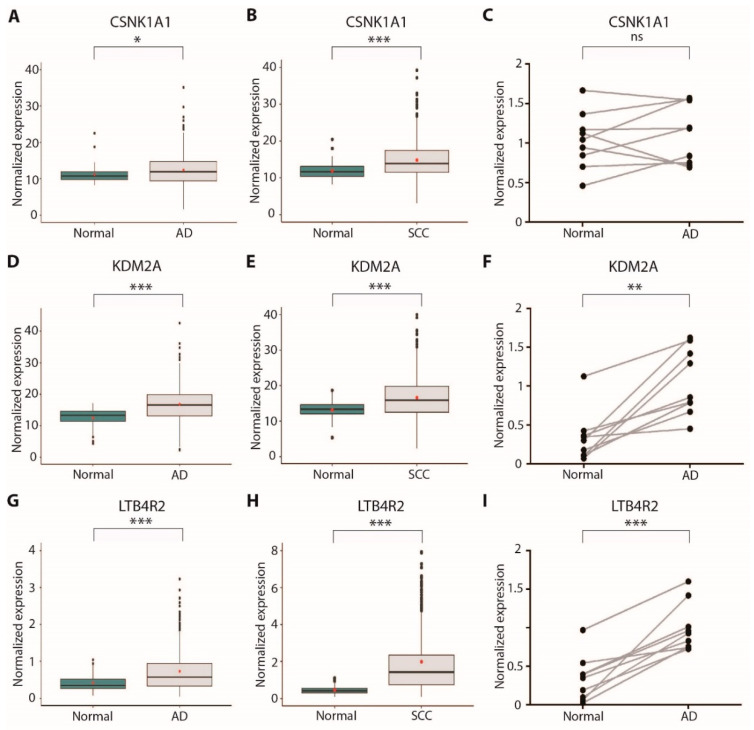
Expression analysis of new druggable dependency genes in lung cancer patients: (**A**) CSNK1A1 expression in TCGA lung adenocarcinoma cohort samples (n = 572, normal = 59, tumor = 513), measured by RNA-Seq; (**B**) CSNK1A1 expression in TCGA lung squamous-cell carcinoma cohort samples (n = 550, normal = 49, tumor = 501), measured by RNA-Seq; (**C**) CSNK1A1 expression in a set of surgical samples from our institute’s biobank (n = 9), measured by RT-qPCR; (**D**) KDM2A expression in TCGA lung adenocarcinoma cohort samples (n = 572, normal = 59, tumor = 513), measured by RNA-Seq; (**E**) KDM2A expression in TCGA lung squamous-cell carcinoma cohort samples (n = 550, normal = 49, tumor = 501), measured by RNA-Seq; (**F**) KDM2A expression in a set of surgical samples from our institute’s biobank (n = 9), measured by RT-qPCR; (**G**) LTB4R2 expression in TCGA lung adenocarcinoma cohort samples (n = 572, normal = 59, tumor = 513), measured by RNA-Seq; (**H**) LTB4R2 expression in TCGA lung squamous-cell carcinoma cohort samples (n = 550, normal = 49, tumor = 501), measured by RNA-Seq; (**I**) LTB4R2 expression in a set of surgical samples from our institute’s biobank (n = 9), measured by RT-qPCR; * *p* < 0.05; ** *p* < 0.01; *** *p* < 0.001; ns = not significant.

We reasoned that CSNK1A1, KDM2A, and LTB4R2 dependencies could become therapeutic intervention points in lung cancer, so we proceeded with further characterization. To gain insights into the role of these genes in lung tumorigenesis, we evaluated their expression levels in the TCGA adenocarcinoma cohort [26] and in a set of samples retrieved from the biobank of our institute. All these genes were significantly upregulated in tumor tissue compared to surrounding healthy lung tissue in the cohorts of both AD and SCC patients derived from TCGA, whereas for SCLC patients normal samples gene expression data were not available (Figure 5A–H). In addition, we evaluated the gene expression levels of these three genes in a set of AD patient samples derived from the biobank of our institute. In this set of samples, the differential expression of KDM2A and LTB4R2 only reached statistical significance (Figure 5C,F,I); however, the number of samples we were able to retrieve from the biobank of our institute was very limited (n = 9) compared to the TCGA cohort (n = 572 for AD and n = 550 for SCC), possibly explaining the lack of significance for CSNK1A1 expression in our set of samples. Collectively, these data suggest that sustained or increased expression of these genes may favor lung tumorigenesis.

### 3.5. Validation of CSNK1A1, KDM2A, and LTB4R2 as Novel Therapeutic Targets in Lung Cancer

**Figure 6:**
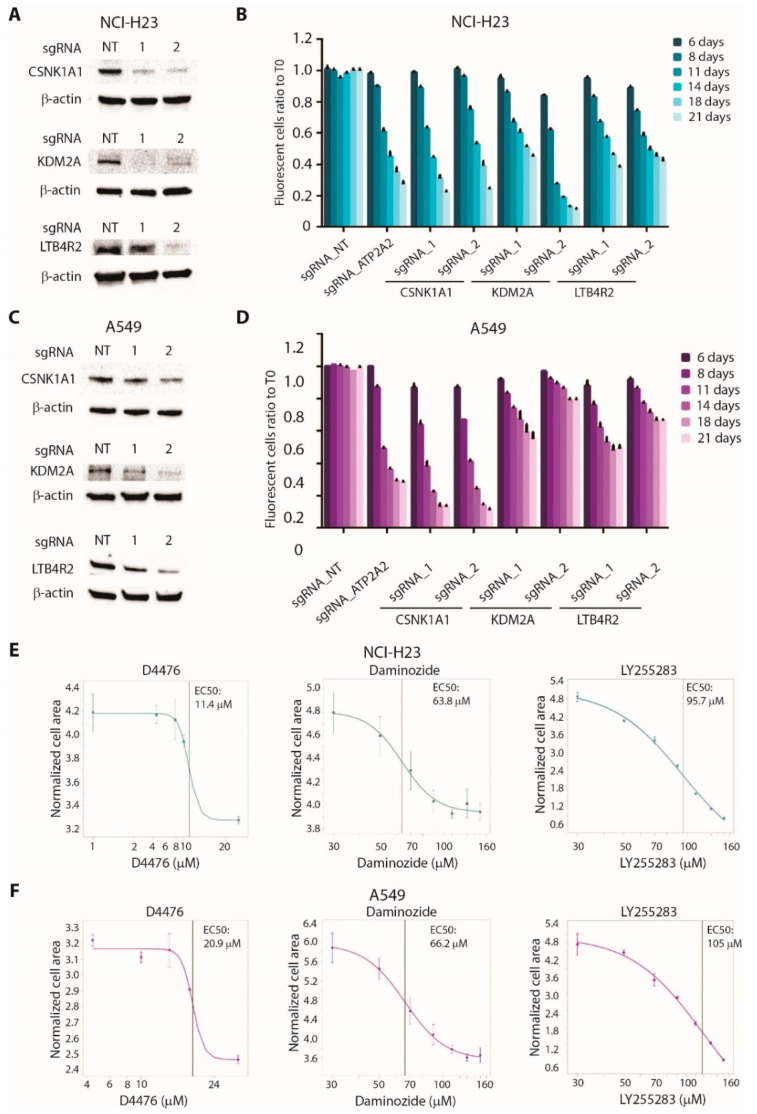
Validation of CSNK1A1, KDM2A, and LTB4R2 as new therapeutic target in lung cancer cell lines: (**A**) Western blot analysis showing CRISPR/Cas9-mediated knockout (KO) of CSNK1A1, KDM2A or LTB4R2 in NCI-H23 cells. Two independent sgRNAs for each gene were used. Anti-β-actin antibodies are used as loading control. (**B**) Competition assay showing reduced proliferation in NCI-H23 cells KO for the indicated target genes. Cells infected with a non-targeting sgRNA (NT) or with a sgRNA for ATP2A2 gene were used as negative and positive controls, respectively. For each time point, the ratio between GFP-positive (infected) and GFP-negative (uninfected) cells was calculated and normalized on T0. Statistical significance was calculated by comparing the normalized ratio for each sample with NT at each time point. Data are expressed as means ± SEM; n = 3. (**C**) Western blot analysis showing CRISPR/Cas9-mediated knockout (KO) of CSNK1A1, KDM2A, or LTB4R2 in A549 cells. Two independent sgRNAs for each gene were used. Anti-β-actin antibodies are used as loading control. (**D**) Competition assay showing reduced proliferation in A549 cells KO for the indicated target genes. Cells infected with a non-targeting sgRNA (NT) or with a sgRNA for ATP2A2 gene were used as negative and positive controls, respectively. For each time point, the ratio between GFP-positive (infected) and GFP-negative (uninfected) cells was calculated and normalized on T0. Statistical significance was calculated by comparing the normalized ratio for each sample with NT at each time point. Data are expressed as means ± SEM; n = 3. (**E**) Sensitivity curves of NCI-H23 cells to CSNK1A1 inhibitor D4476, KDM2A inhibitor daminozide, or LTB4R2 inhibitor LY255283. Data are expressed as means ± SEM; n = 3. (**F**) Sensitivity curves of A549 cells to CSNK1A1 inhibitor D4476, KDM2A inhibitor daminozide, or LTB4R2 inhibitor LY255283. Data are expressed as means ± SEM; n = 3.

To validate the dependency of tumor cells on CSNK1A1, KDM2A, and LTB4R2 in vitro, we generated the knockout (KO) of each gene by independently introducing two sgRNAs in two AD cell lines, NCI-H23 and A549 (Figure 6A,C and Supplementary Figure S1). The KO cell lines of all three genes showed decreased cell proliferation or survival in a competition assay. ATP2A2, being a strongly essential gene, was used as the positive control (Figure 6B,D).

To provide an in vitro validation of the possibility of targeting these genes with chemical inhibitors, we treated NCI-H23 and A549 cell lines with the CSNK1A1 inhibitor D4476, the KDM2A inhibitor daminozide, or the LTB4R2 inhibitor LY2552843. As shown in Figure 6E,F, all three compounds showed inhibitory effects on cell proliferation, with EC50 values in the micromolar range. Notably, D4476 was the most effective in reducing cell proliferation, in accordance with the strong sensitivity to the KO of CSNK1A1 observed in both cell lines (Figure 6B,D).

These data suggest that CSNK1A1, KDM2A, and LTB4R2 may be considered relevant therapeutic targets in lung cancer, warranting further investigations on the roles of these genes in different lung cancer histological subtypes.

## 4. Discussion

In this work, we identified new possible therapeutic targets in lung cancer, combining in vitro results produced in our laboratory with large collections of data available in public repositories. A great part of this analysis was conducted on dependency data generated by our CRISPR/Cas9 screening, together with data provided by the DepMap consortium. This approach allowed us to identify genes and pathways that represent vulnerabilities in different lung cancer histotypes. Each list of genes was crossed with the Drug–Gene Interaction Database to select dependency genes that can be targeted with inhibitory chemical compounds. From this analysis, we identified drugs that are used in clinical practice for lung cancer, such as irinotecan and gemcitabine [27,28], as well as a large list of compounds whose interactions with gene targets have already been characterized and that are currently under evaluation in clinical trials [29,30,31]. These results strongly support the validity of our approach. In addition, this strategy allowed us to identify three target genes that are poorly characterized and still require biological validation in lung cancer: CSNK1A1, KDM2A, and LTB4R2.

The CSNK1A1 gene encodes the alpha isoform of casein kinase 1 (CK1α), a serine–threonine kinase that plays a critical role in regulating WNT/β-catenin signaling via the phosphorylation of multiple pathway components with both positive and negative consequences on overall signal transduction [32]. In addition, CK1α is involved in other oncogenic pathways, including autophagy and p53 and NFkB signaling, having either pro-oncogenic or tumor suppressor functions, depending on the context [33,34]. Several CK1 small-molecule inhibitors have been developed, showing different specificities and promising results in preclinical studies, but none has been included in clinical trials so far [35]. Our results showed that CSNK1A1 gene is a vulnerability in lung AD, indicating that this gene has a predominant pro-oncogenic function in this setting and supporting the possible use of pharmacological inhibitors in clinical trials.

LTB4R2 encodes the leukotriene B4 receptor 2, a G-protein-coupled receptor regulating chemotaxis and wound healing. In addition to inflammatory processes, this receptor has been shown to be implicated in invasion and metastatic colonization of lung and other cancers [36,37,38]. In this work, we found LTB4R2 to be a druggable essentiality gene in SCC, whereas in a previous work we found LTB4R2 to be a vulnerability specifically associated with the presence of KRAS mutation in lung AD [9]; thus, the sensitivity to the KO of this gene may be context-specific and not limited to a single lung cancer subtype.

KDM2A is a histone demethylase that specifically removes methyl residues from lysine 36 of histone H3, promoting a chromatin-repressed state [39,40]. KDM2A has been demonstrated to promote tumorigenesis in different cancer settings, including lung cancer [41,42]. The possibility to pharmacologically hit KDM2A was demonstrated by the discovery that the plant growth modulator daminozide is a selective inhibitor of the KDM2/7 demethylase subfamily [43]. These findings, together with our results, suggest that further development of KDM2A inhibitors may be a feasible path for the realization of novel therapeutic strategies.

Importantly, we found equal dependency on KDM2A in AD and SCLC cell lines, highlighting the importance of this gene and suggesting the possibility to also develop a targeted therapy approach for SCLC patients, whose therapeutic options are still very limited.

## 5. Conclusions

Overall, in our study we described the use of an innovative integrative genomic approach to identify new candidate genes for lung cancer treatment, namely CSNK1A1, KDM2A, and LTB4R2. Further experiments are required to define each gene contribution to tumor development and to characterize the specific mechanisms associated with cancer dependency. Future evaluation is foreseen to fully validate CSNK1A1, KDM2A, and LTB4R2 as druggable candidates in clinical practice.

## Data Availability

The datasets used or analyzed during the current study are available from the corresponding author on reasonable request.

## Supplementary Materials

The following are available online at https://www.mdpi.com/article/10.3390/cancers13143477/s1: Figure S1: Evaluation of CRISPR/Cas9-mediated genetic modification. Table S1: Complete list of enriched pathways in AD, SCLC, or SCC dependency genes. Table S2: Complete list of druggable targets within dependency genes lists in AD, SCLC, and SCC. Table S3: Features of patients analyzed by RT-qPCR. Table S4: Sequences of sgRNAs and primers. Video S1: CRISPR/Cas9 screening for essentiality genes.
