# Supplementary material for: CSNK1A1, KDM2A, and LTB4R2 Are New Druggable Vulnerabilities in Lung Cancer
Source: Cancers (Basel). 2021 Jul 12;13(14):3477. doi: 10.3390/cancers13143477 (PMC8305418; doi:10.3390/cancers13143477)
Supplement: Supplementary file 1 [file cancers-13-03477-s001.zip › Supplementary files/Supplementary Figure1.pdf]

SUPPLEMENTARY FIGURE 2

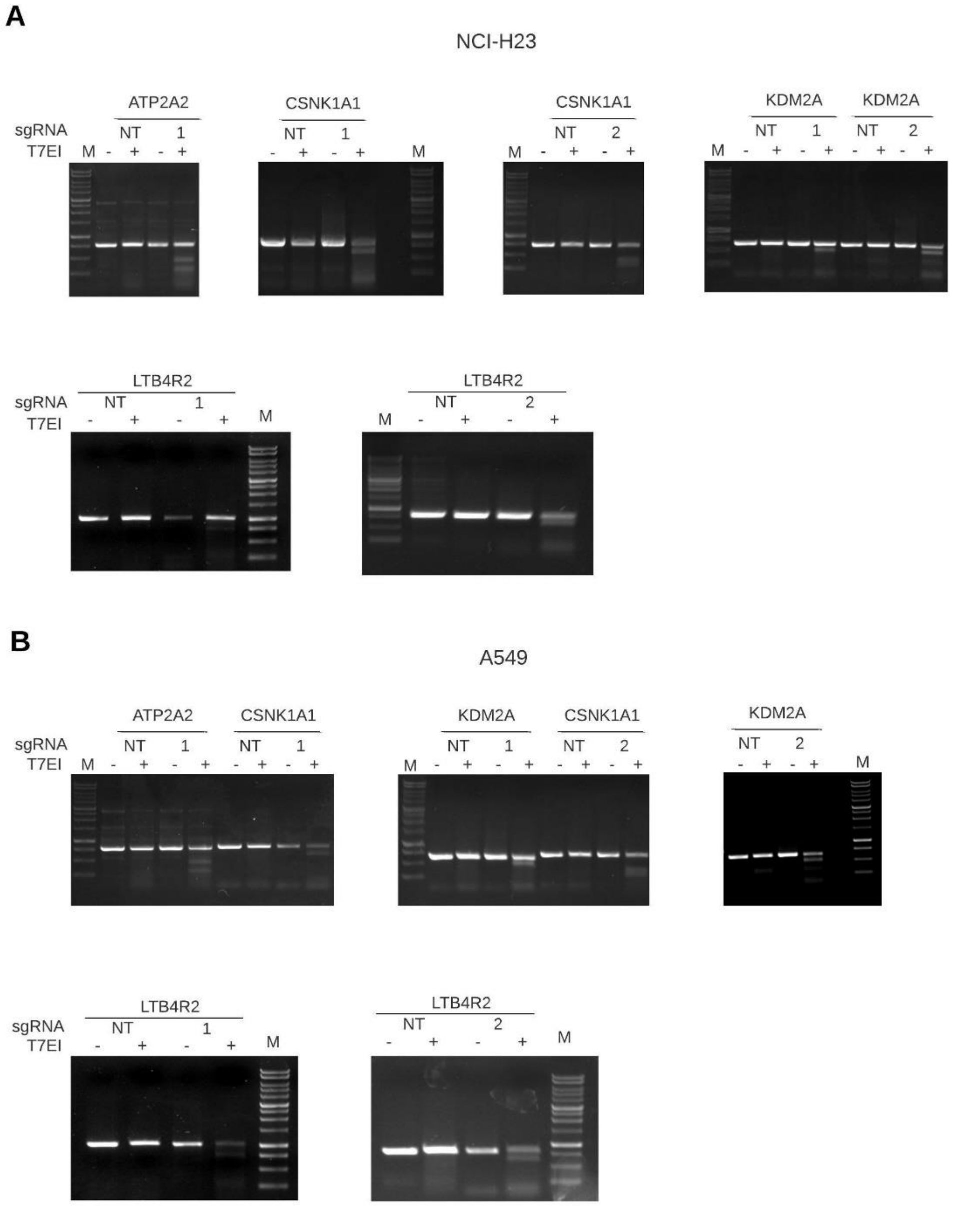

Figure S1: Evaluation of CRISPR/Cas9-mediated genetic modification

A) Agarose gel runs showing Alt-R mismatch analysis in NCI-H23/Cas9 cells infected with the indicated sgRNAs. As negative control (CT), NCI-H23/Cas9 cells infected with a non-targeting (NT) sgRNA were used. The addition of T7 endonuclease I (T7EI) is indicated over each lane. M indicates a molecular weight marker in the range of 100bp-1kb. B) Agarose gel runs showing Alt-R mismatch analysis in A549/Cas9 cells infected with the indicated sgRNAs. As negative control (CT), A549/Cas9 cells infected with a non-targeting (NT) sgRNA were used. The addition of T7 endonuclease I (T7EI) is indicated over each lane. M indicates a molecular weight marker in the range of 100bp-1kb.
